# Mr. Marsh, on the Deleterious Effects of the Lolium

**Published:** 1799-07

**Authors:** Richard Marsh

**Affiliations:** Surgeon, (2d Wilts. Militia.) Plymouth-Dock


### *To the Conductors of the Medical and Physical Journal.*

Gentlemen,

I Have taken the liberty of sending you the inclosed account of the *deleterious effects of the lolium, or darnel,* which, if you should think worthy of insertion in your useful publication, it is my wish, that it may be so; at the same time I have to lament, that the progress of the poison was not taken down so minutely as it would have been, had I at the time entertained the least thought of its being exhited to the public eye, but the authenticity of the facts may be depended on. It is also a circumstance of some concern to me, that it should not have fallen to the lot of some person, possessing greater abilities than myself, to have noticed the fact, that darnel produces effects extremely inimical to the animal economy; but as I entertain great hopes that the present hints may call forth those abilities, I consider it a duty incumbent on me, to mention the following, among many others that have occurred in the course of a practice of a few years in a partnership at Highworth, Wilts, with my brother-in law, Mr. Powell, to whom I am obliged for the succesful mode pursued in several cases; and as in the northern part of the county of Wilts the darnel much abounds, and the poorer class eat it with that part of the corn called tarling corn, I have hopes that a more succesful mode of treatment may be devised, which may save a numerous and well-deserving part of the community from great pain and torture.

I remain,

Your obedient humble Servant,

Plymouth-Dock,

*June* 12, 1799,

RICHARD MARSH,
Surgeon, (2d Wilts. Militia.)

In the month of September a sack of leased wheat with an equal quantity of tarling wheat (*i. e.* the refuse seeds which pass the sieve) abounding very much with darnel (lolium), which by the generality of people, where the plant is much known, is called *cheal,* were ground and dressed together, and in the evening about ten o'clock, bread was made of a part of it. Of this bread, James Edmonds, about thirty-three years of age, and Robert his son, aged thirteen, eat the next morning about three o'clock; at five (two hours after), James became sick and giddy, vomited and purged much, felt pain and tightness in the calves of his legs, was confined at home the whole day, but on the following day was so far recovered, as to be able to resume his work. Robert eat, during the day, about a pound and a half of this bread, and at night on his return from his work, he eat more of the same; he fell giddy, and had pain of the head during during the whole of the firft day, with great pain and tightnefs of the legs, efpecially of the calves of the legs, extending to the ancles, attended with rednefs and fwelling, and itching of the fkin, but it did not vomit or purge him till the third day. James, eleven years old—John, three—and Elizabeth four,—all partook of this bread the following morning about nine o'clock. They foon became giddy, were fick, vomited and purged greatly, their legs became painful, felt exceffive tight, were fwelled, inflamed, and itched much, and continued in that ftate eight or nine days, when the fymptoms gradually difappeared, producing in one of them only (James) a fmall collection of a gelatinous fluid, in the infide of the foot. But with Robert who eat with his father at three o'clock in the morning, and alfo in the evening, and who was not vomited and purged till the third day, the pain and inflammation continued to increafe, till it terminated in gangrene; fphacelous fucceeded, and he was under the neceffity of fuffering amputation of both legs. Very little general fever accompanied this till the latter ftage of the difeafe, which it is prefumed, was the effect of abforption. The remedies made ufe of in this cafe (and that too without any fenfible advantage) were, in the beginning, evacuants, in the latter ftage, camphor and bark, with the ufe of fpirituous fomentations and antifceptic cataplafms. It fhould however be remarked, that this poor family lived at feven or eight miles from medical affiftance, and therefore they were not attended till two or three days after their attack.

In feveral cafes which have fince occurred, as foon as the legs became painful, attended with inflammation and fwelling, fcarifications were made of confiderable length and depth, which, with evacuants in the very firft ftage, and afterwards large dofes of camphor, with nitre and opium occafionally, have been attended with fuccefs.

It may perhaps be worthy of notice, to remark that this plant feems to have produced more deleterious effects when eaten quite new and warm, which was the cafe with James and Robert, at three o'clock in the morning, and there is no doubt but the father would have fuffered equally with the fon, had it not fo foon been thrown off the ftomach. There is alfo another circumftance to be noticed, which is, that all the patients I have feen have univerfally complained of violent pain in the calves of their legs, and expreffing their pain nearly fimilar, viz. as though their calves were very tightly bound with cords.

